# Biochemical Characterization of Medaka (*Oryzias latipes*) Transglutaminases, OlTGK1 and OlTGK2, as Orthologues of Human Keratinocyte-Type Transglutaminase

**DOI:** 10.1371/journal.pone.0144194

**Published:** 2015-12-29

**Authors:** Ayaka Kikuta, Eri Furukawa, Ryota Ogawa, Natsuki Suganuma, Mai Saitoh, Toshiyuki Nishimaki, Takafumi Katsumura, Hiroki Oota, Tadafumi Kawamoto, Hideki Tatsukawa, Hisashi Hashimoto, Kiyotaka Hitomi

**Affiliations:** 1 Graduate School of Pharmaceutical Sciences, Nagoya University, Nagoya, 464–8601 Japan; 2 Kitasato University School of Medicine, Sagamihara, 252–0734 Japan; 3 Radioisotope Research Institute, School of Dental Medicine, Tsurumi University, Tsurumi, Yokohama, 230–8501 Japan; 4 Bioscience and Biotechnology Center, Nagoya University, Nagoya, 464–8601 Japan; University of Maryland School of Medicine, UNITED STATES

## Abstract

Calcium-dependent transglutaminases (TGs) are a family of enzymes that catalyze protein cross-linking and/or attachment of primary amines in a variety of organisms. Mammalian TGs are implicated in multiple biological events such as skin formation, blood coagulation, and extracellular matrix stabilization. Medaka (*Oryzias latipes*) has been used as a model fish to investigate the physiological functions of mammalian proteins. By analysis of the medaka genome, we found seven TGs orthologues, some of which apparently corresponded to the mammalian TG isozymes, TG1, TG2, and Factor XIII. All orthologues had preserved amino acid residues essential for enzymatic activity in their deduced primary structures. In this study, we analyzed biochemical properties of two orthologues (OlTGK1 and OlTGK2) of mammalian epithelium-specific TG (TG1) that are significantly expressed at the transcriptional level. Using purified recombinant proteins for OlTGK1 and OlTGK2, we characterized their catalytic reactions. Furthermore, immunohistochemical analyses of fish sections revealed higher expression in the pancreas (OTGK1), intervertebral disk (OlTGK2) and pharyngeal teeth (OlTGK2) as well as in the skin epidermis.

## Introduction

Transglutaminases (TGs) are the enzymes that catalyze formation of isopeptide-bonds between glutamine and lysine residues of their substrate proteins in a calcium-dependent manner [1–2]. In addition to lysine residues, primary amines (e.g., polyamine) and water molecules can also react with glutamine residues, resulting in the attachment of the amine and conversion of the glutamine to a glutamic acid residue, respectively. In mammals, these enzymatic post-translational modifications are observed in multiple biological processes such as blood coagulation, skin formation, extracellular matrix stabilization, apoptosis, and also with non-catalytic functions [3]. In humans, these catalytic reactions are conducted in several tissues and cells by TGs family members comprising eight isozymes; Factor XIII and TG1-TG7. Because the physiological roles of TGs are diverse, their complete characterization remains incomplete.

Among the tissues, skin formation is a prominent target of TG studies because the cross-linking reaction products formed in epidermal keratinocytes clearly contribute to their integrity and barrier function [4–6]. Skin-type TGs (TG1, TG3, and TG5) have been reported to be responsible for the cooperative formation of these cross-links. In our recent studies, TG6, mainly expressed in neuronal cells, also appeared to have enzymatic activity in the epidermis [7]. Furthermore, TG1 is also expressed in other epithelial tissues during development and in the matured various tissues in mice [8, 9]. Thus, studies on the physiological significance of skin-type TGs during the epidermis formation have advanced through several aspects of biochemical characterization of these TGs and through use of knockout mouse [10, 11]. However, cooperative cross-linking of their epidermal substrates and functional expression in tissues other than the skin remain still unclear for these isozymes.

In recent years, small fish such as zebrafish (*Danio rerio*) and medaka (*Oryzias latipes*) have been used for several studies, including investigation of the mechanisms of basic biological phenomena, drug screening, and phenotype analysis of diseases [12–15]. These species have advantages, such as short generation time, high fertility, and low maintenance cost. In particular, the reverse-genetic approach to knock down a specific gene is more feasible in these organisms than in mammals. Characterization of the TG orthologues of zebrafish has recently been reported: Twelve TG orthologues exist in this organism and some appear to be responsible for bone formation and signal transduction [16, 17].

In this study, we targeted the medaka, as alterative model fish, to discern the expression patterns and physiological functions of skin-type TGs, particularly TG1. Because TG1 is a major skin enzyme, knockout mice die after birth due to aberrant skin formation and damages in other tissues. Although it is possible to establish a mouse with tissue-specific ablation of TG1 expression, we considered that medaka might be suitable for studies on loss of function. However, information about biochemical properties of medaka TGs and their functional roles in skin formation and/or other tissues has not been reported to date. Therefore, we attempted to investigate the genes encoding medaka TGs exhaustively and to biochemically medaka TGs using recombinant proteins.

Upon analysis of the medaka genome for orthologues of mammalian TG1, all the genes related to mammalian TG isozymes were analyzed. Then, we biochemically characterized gene products of the medaka TG1-orthologue genes and conducted immunohistochemical analyses using isozyme-specific antibodies. Two enzymes that were apparent TG1 orthologues appeared to localize to the epidermis, as they do in mammals. However, unexpectedly, they were expressed in other tissues, such as the salivary gland, invertebrate disk, and pharyngeal teeth in addition to the skin. A series of finding provided information on possible novel functions of TGs and their applications.

## Materials and Methods

### Ethic statement

Animal care and experiments were carried out according to the Regulations for Animal Experiments in Nagoya University. This study was approved by the Animal Care and Use Committee of Nagoya University.

### Fish experimental conditions

Medaka fish (Cab strain) was obtained from National BioResources Project of Japan (NBRP) (Okazaki, Japan). The fish used in this study were maintained at the following housing conditions: A 16-L tank with a water circulating system and feeding every day, at 26°C, under a 14 h light /10 h dark cycle, by 20–25 fish per tank. Surgery was performed under anesthesia using MS-222 (tricaine methane sulfonate; Sigma-Aldrich, St. Louis, MO), and all efforts were carefully made to minimize suffering.

### Sequence analyses of medaka orthologues

Based on the similarity with mouse TGases family member, homologous sequences for possible medaka transglutaminase cDNAs were searched (see Results) using the database from the NBRP. Briefly, using the database on NBRP, orthologues were searched the cDNAs as query of mouse TGs (FXIII, TG1-TG7).

Plasmid harboring each cDNA was obtained from NBRP or by cloning in our laboratory as follows. Among the cDNAs for orthologues in medaka, one apparent homologous sequence to mammalian skin type TG (TG1) was designated as OlTGK2 (OlTGK2; olsp48d24). As the other orthologues, each cDNA clone was obtained from NBRP (clone name: OlTGB; olsp35n14, OlTGT; olsp62a16, OlTGF olec36d21, OlTGO; olec36g08). Since the sequence of the full-length cDNA had not been available, we clarified them using the provided plasmid vectors harboring each cDNA. By further investigation, genes for orthologues as OlTGK1 and OlTGK3 were found on the database of genome (Ensemble: http://www.ensembl.org/index.html). Hence, for both genes, using embryonic medaka cDNA library, cDNAs were cloned using information on the genome database and was characterized for possible open reading frame. Sequences of cDNAs were analyzed with an automated fluorescent sequencer, ABI PRISM 310 (PE Applied Biosystems, CA). Then, these sequence data have been deposited to DDBJ (DNA Data Bank Japan). For alignment and construction of phylogenetic tree with human TGs, software (Genetyx ver. 11.0) was used based on these amino acid sequences.

### Bacterial expressions of the recombinant proteins for OlTGK1 and OlTGK2

In order to express recombinant proteins for OlTGK1 and OlTGK2, using the cDNA as a template, each cDNA fragment attaching with the appropriate restriction enzyme sites at both sides was amplified by the specific primers. For the expression of recombinant protein in an IPTG (isopropyl β-D-thiogalactoside)-inducible manner, the bacterial expression vector, pET24d (Novagen-Merck) was used which had been modified to attach a hexahistidine-tag at the N-terminus upon expression. The DNA fragment encoding for OlTGK1 or OlTGK2 was inserted into the expression vector using each specific restriction enzyme site. The host bacterial strains, BL21(DE3)LysS was transformed with the expression vector plasmid. To express of the recombinant proteins for other orthologues of the medaka TGs (OlTGB, OlTGT, OlTGO, and OlTGF), the same strategy with the pET vector system was used with BL21(DE3)LysE or BL21(DE3)LysS as hosts (unpublished data).

### Bacterial expression and purification of recombinant proteins

The resulting transformant was grown in the medium (L-broth) until log-phase at 37°C, and then induced for expression by adding IPTG (1 mM) at 25°C for 3–6 hrs or more. The grown bacteria was harvested by centrifugation and lysed with a hypotonic buffer (10 mM Tris-Cl, pH 8.0, 150 mM NaCl, 1mM PMSF, benzamidine, and 1 mM β-mercaptoethanol). The supernatant was obtained by centrifugation (12,000 g x 5 min) twice and then subjected to metal-affinity chromatography (TALON; Clontech-TAKARA, Kyoto, Japan). To further purify the recombinant protein, the eluted fractions from the affinity-chromatography were subjected to size fractionation chromatography (Superdex-200 increase; GE Healthcare Bio-Sciences AB Uppsala, Sweden) in a PBS buffer containing 1 mM β-mercaptoethanol and 1 mM EDTA.

### Assay of the enzymatic activity of the recombinant protein

For evaluation of the enzymatic activity of the recombinant protein, a plate assay was carried out in the well of the 96 multitire plate coated with β-casein [18]. Briefly, the purified recombinant protein was incubated with biotin-labeled pentylamine (bio-Cd) (EZ-link^TM^ 5-(biotinamido)pentylamine (Pierce, IL) in an appropriate Tris-Cl buffer (pH 8.0) containing CaCl_2_. The reaction mixture was incubated at 25°C for the indicated times and the reaction was stopped by the addition of excess EDTA. After washing several times with TBS buffer containing 0.1% tween-20, the incorporated bio-Cd into coated β-casein was measured using streptavidin-conjugated peroxidase and the peroxidase substrate 3, 3’, 5, 5’-tetramethylbenzidine (Sigma).

### Preparation of specific antibodies and immunochemical analyses

Rabbit polyclonal anti-OlTGK1 and anti-OlTGK2 sera were prepared by Japan Lamb (Hiroshima, Japan) using the purified recombinant proteins for OlTGK1 and OlTGK2 as antigen. The IgG fractions in the serum were affinity-purified using NHS-activated Sepharose 4 Fast Flow (GE Healthcare Bio-Sciences), which had been immobilized with each recombinant protein.

For western blotting, the protein samples were subjected to 7.5% SDS-polyacrylamide gel electrophoresis (SDS-PAGE) and then transferred to the loaded proteins onto PVDF membrane (Millipore Merck, Darmstadt, Germany) by a standard method. After blocking with skimmilk, the membrane was incubated with polyclonal antibody solution at 37°C for 1 hr. Signals were obtained using the secondary antibody conjugated with peroxidase and then developed by chemiluminescence reagent (Thermo scientific, Rockford, IL).

Immunohistochemical analysis was performed using 5 μm paraffin section of medaka. The paraffin-fixed sections were prepared by standard method following dewax by xylene and ethanol. Non-fixed frozen sections were prepared with an adhesive film and a disposable tungsten carbide blade, Cryofilm type 2C (9) and SL-T30 (UF), respectively, (SECTION LAB Co. Ltd., Japan) according to the method described by Kawamoto et al. [19]. After blocking the endogenous peroxidase with hydrogen peroxide, preparation was treated with primary antibody. After washing, the samples were subjected to the ABC kit (Vectastain) consisting of biotin-conjugated secondary antibody, avidin, biotin-conjugated peroxidase. Then the samples were developed with diaminobenzidine.

## Results

### Genetic analysis of medaka orthologues to mammalian TGs

**Fig 1:**
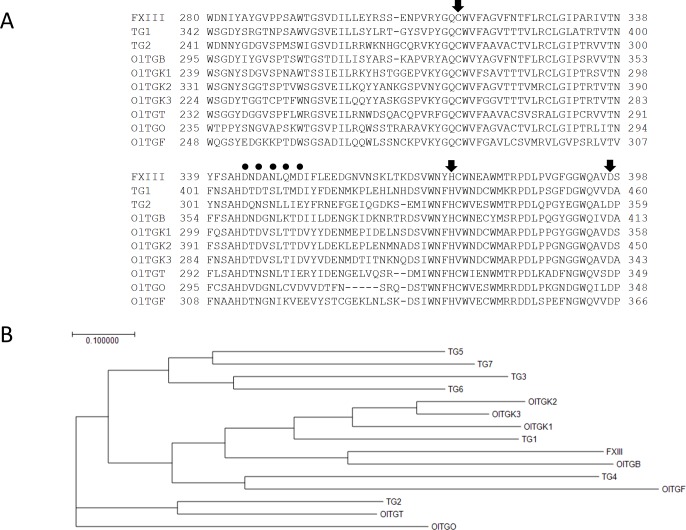
Sequence alignment of human TGs and medaka orthologues and phylogenic tree based on primary structure. Sequence alignment around the catalytic triad including Cys (the active-site), His, and Asp was shown with respect to the major human TGs and the medaka orthologues: Human TG1 (NP_000350), TG2 (NP_004604), FXIII (NP_000120), as well as medaka orthologues. The catalytic triad, containing the active site Cys and the other two amino acid residues (Asp, His) is indicated by arrows. The possible residues coordinating calcium ion are indicated as circles. The numbers of the amino acid residues are described with the sequences. (A). The phylogenic tree was constructed (1000 boostrap trials, Neighbor-Joining Method plot) based on the deduced amino acid sequences for human FXIII, TG1, TG2, TG3 (NP_003236), TG4 (NP_003232), TG5 (NP_963925), TG6 (NP_945345), TG7 (NP_443187), and their medaka orthologues. Each cDNA sequence of medaka orthologues has been deposited to DDBJ (DNA Data Bank Japan) as following accession numbers: OlTGB (LC068825), OlTGT (LC068826), OlTGK1 (LC068829), OlTGK2 (LC068830), OlTGK3 (LC068831), OlTGF (LC068827), and OlTGO (LC068828) (B).

No information about medaka TGs as orthologues of mammalian enzyme family members has been reported so far. Initially, we searched the database of medaka cDNA (NBRP) with a query containing the full-length cDNAs for mouse FXIII, and TG1-TG4, which are the major TG isozymes. Several cDNA sequences showing significant homology were found in each search of the medaka database and also in Ensembl genome database. From the NBRP cDNA clone bank and also embryonic cDNA library, these OlTGs cDNA encoding seven orthologues as designated, OlTGB, OlTGK1, OlTGK2, OlTGK3, OlTGT, OlTGO, and OlTGF, were revealed. All the nucleotide sequences encoding open reading frame were analyzed and deposited to DDBJ (Fig 1).

Among these, the cDNA sequence of OlTGB (possible orthologue to human FXIII) was identical with that of emTGase (embryonic TGase) previously cloned from medaka egg [20].

By the alignment, the deduced amino acid sequences showed significant homology to human TGase family members, and their calculated molecular size were similar to those for other vertebrate TGs. Several characteristics of TGs were preserved in the deduced primary structure. As shown in Fig 1A, the catalytic triad, which includes the cysteine residue essential for transamidation and also possible calcium-binding sites were conserved in all of the OlTGs [21]. Furthermore, the other regions were similar to human TGs, which indicates there are four possible domains that are characteristic of mammalian TGs: a β-sandwich, catalytic core, a β-barrel 1, and β-barrel 2.

Then, based on the alignment of the predicted amino acid sequences, we constructed a phylogenic tree, that revealed the presence of the apparent orthologues for FXIII (OlTGB), TG1 (OlTGK1, OlTGK2, and OlTGK3), TG2 (OlTGT), and TG4 (OlTGF) (Fig 1B). In this family, three orthologues corresponding to mammalian TG1 existed. While OlTGO did not apparently coincide with any mammalian TG isozymes, OlTGF is located in a position relatively close to that of TG4.

### Expressions and purifications of recombinant proteins for OlTGK1 and OlTGK2

By the alignment, in medaka, three orthologues were found but, in this report, we have shown the results of orthologues (OlTGK1 and OlTGK2) of mammalian major skin TG, TG1. When we attempted to amplify the cDNA fragment corresponding to OlTGK3 using reverse-transcription polymerase chain reaction (RT-PCR) of whole fish body RNA, much less products was obtained probably due to lower amounts of mRNA in the skin (S1 Fig). Hence, we focused on OlTGK1 and OlTGK2 as skin-type TGs in medaka. The nucleotide sequences of OlTGK1 and OlTGK2 show open reading frame of 2067 and 2340 bps corresponding to 688 and 778 amino acids, respectively.

**Fig 2:**
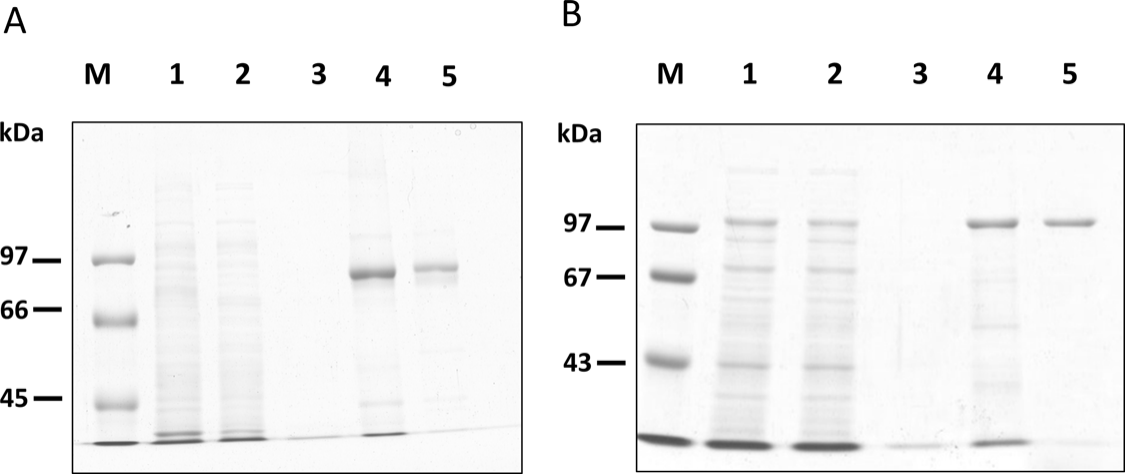
Purification of OlTGK1 and OlTGK2 recombinant proteins produced in bacteria. The soluble fraction containing recombinant OlTGK1 (A) and OlTGK2 (B) proteins were purified using metal ion affinity-gel and size exclusion chromatography. Each sample was subjected to 7.5% SDS-PAGE following Coomassie Brilliant Blue staining: lane 1; applied samples for affinity chromatography, lane 2; flow-through fractions, lane 3; washed fractions, lane 4; eluted fractions, lane 5; the peak fractions from the size separation performed using Superdex-200 increase. M: molecular mass marker.

In order to biochemically characterize OlTGK1 and OlTGK2, both recombinant proteins were produced in bacteria as hexahistidine-tagged proteins for purification. Inducible expression was initially carried out using a standard cultivation procedure at 37°C. Both OlTGK1 and OlTGK2 were expressed in bacteria in significant quantities, and both proteins were of the expected size. However, substantial amounts of the expressed proteins were present as insoluble proteins in both cases. Hence, proteins were induced induction at lower temperature (26°C), which resulted in successful production of soluble recombinant proteins (S2 Fig; OlTGK2). The harvested soluble fraction of each protein was affinity-purified and further purified by size-exclusion chromatography (Fig 2). Finally, the most homogeneous fractions were obtained in great enough amounts for biochemical analysis and antibody production.

### Evaluation of the enzymatic activity of the recombinant OlTGK1 and OlTGK2

**Fig 3:**
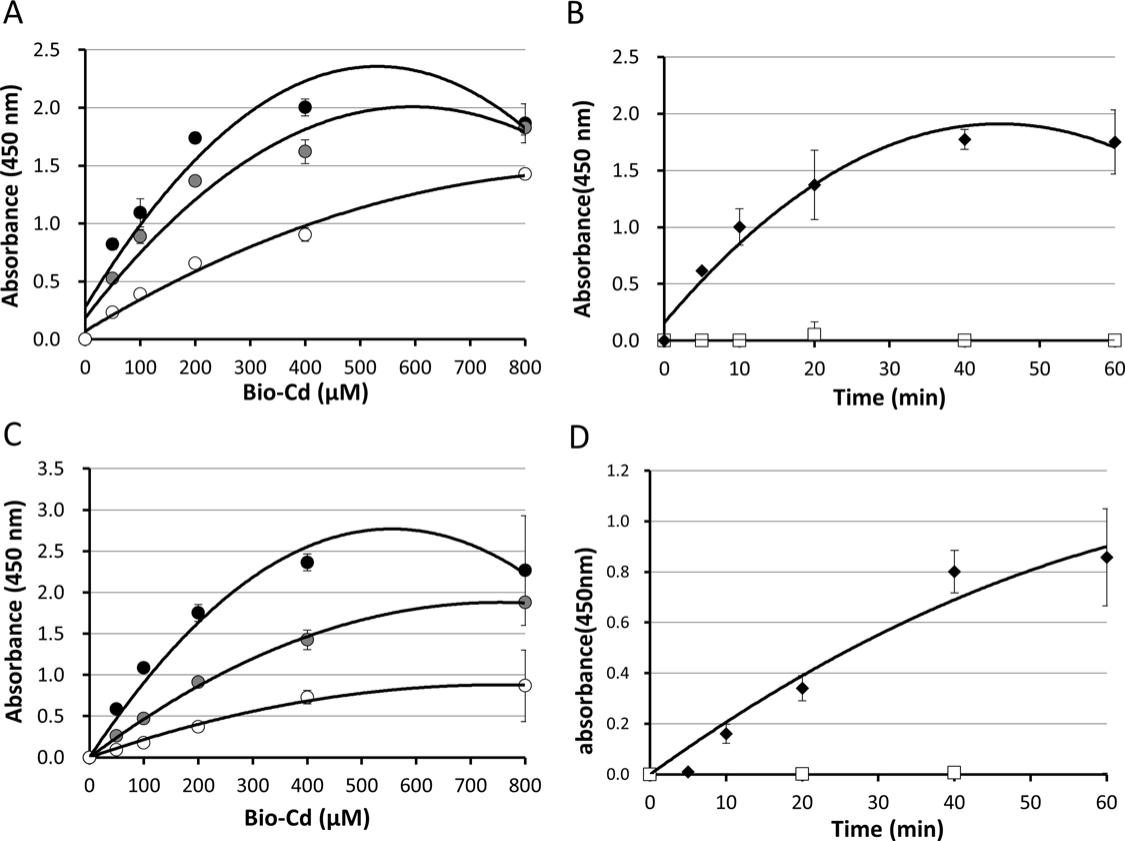
The enzymatic activities of OlTGK1 and OlTGK2 evaluated by incorporation of biotin-cadaverine. The transamidating activities of the purified recombinant OlTGK1 and OlTGK2 proteins were measured by incorporation of biotinylated cadaverine (bio-Cd) into β-casein coated onto wells of microtiter. Incorporation was measured at various concentrations of bio-Cd and with different amounts of the enzymes for 20 min: For OlTGK1 (A), the white, gray, and black circles indicate 2, 5, and 10 ng/μl (final concentrations) of proteins, respectively. For OlTGK2 (C), the white, gray, and black circles indicate 2, 5, and 20 ng/μl of proteins, respectively. Time course reactions (0 to 60 min) with 2 ng/μl of enzyme proteins in the absence (black) and presence (white) of EDTA were shown (B: OlTGK1, D: OlTGK2).

In order to characterize the enzymatic activities of these enzymes, the recombinant proteins were analysed by incorporation of bio-Cd into a glutamine-donor substrate coated onto a microtiter plate. As shown in Fig 3A and 3C, apparent incorporation was observed in the presence of three-different amounts of OlTGK1 and OlTGK2 (2–20 ng/ μl), in a substrate concentration-dependent manner. Furthermore, time-dependent incorporations were also observed in both TG reactions (Fig 3B and 3D). No reaction products were observed in the presence of EDTA, indicating that the medaka TGs are also calcium-dependent enzyme, as are the mammalian TGs.

### Immunohistochemical analyses of OlTGK1 and OlTGK2

To analyze the expression patterns of both OlTGKs in the medaka body, polyclonal antibodies were prepared using standard methods. In order to avoid cross-reaction with other medaka TGs, the polyclonal sera were affinity-purified using columns containing immobilized OlTGK1 and OlTGK2 to obtain each specific antibody. The quality of the affinity-purified antibody fractions was confirmed by immunoblotting using other recombinant proteins as antigens. No cross-reactivity was observed (S3 Fig), demonstrating that each antibody is quite specific to the desired isozyme. In particular, no cross-reaction was confirmed between close paralogue, OlTGK1 and OlTGK2.

**Fig 4:**
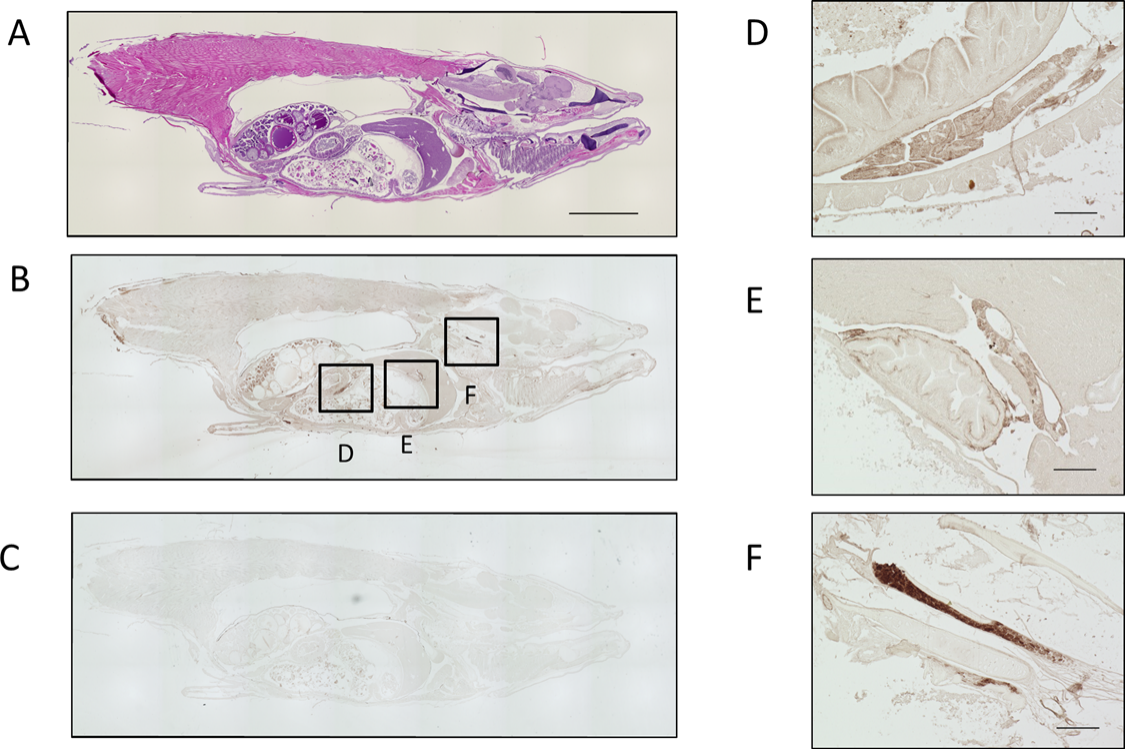
Immunohistochemical analysis of OlTGK1 using medaka sections. Whole body sections of medaka fixed with methanol and acetic acid following by paraffin were analyzed by immunostaining. The serial sections were stained with hematoxylin and eosin (HE) (A) and subjected to immunoreaction with an affinity-purified polyclonal antibody against OlTGK1 (B) as well as a rabbit immunoglobulin solution at a similar concentration (C). The whole body image (B) around the stained area was enlarged; pancreas (D), Brockmann body (E), and salivary gland (F). The scale bars indicate 2 mm (A) and 100 μm (D-F).

**Fig 5:**
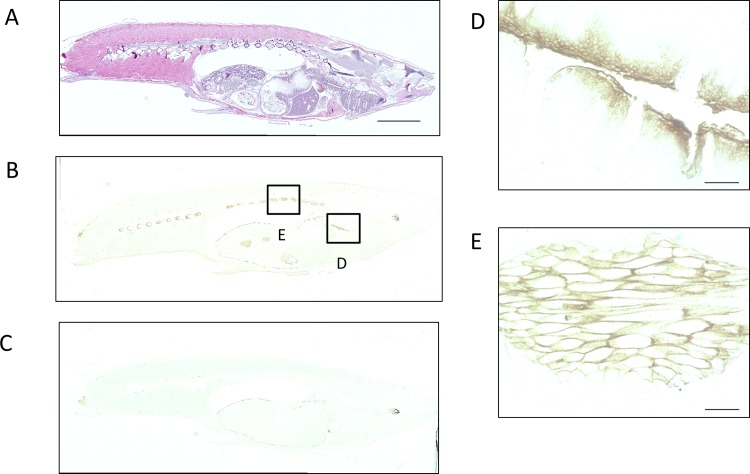
Immunohistochemical analysis of OlTGK2 using medaka sections. Whole body sections were used for immunohistochemical analyses as described in the legend to Fig 4. HE staining was carried out in the whole body, with serial section and paralleled. The serial sections were stained with HE (A) and subjected to immunoreaction with an affinity-purified polyclonal antibody against OlTGK2 (B) and as well as a rabbit immunoglobulin at a similar concentration (C). The image at the stained area was enlarged: the pharyngeal teeth (D) and intervertebral disk (E). The scale bars indicate 2 mm (A) and 50 μm (D, E).

Next, the paraffin-fixed medaka tissue sections were immunostained using the purified antibodies. Representative patterns of OlTGK1 and OlTGK2 expression are shown in Figs 4 and 5, respectively. Signals corresponding to OlTGK1were observed in the whole body, but also significantly in the following sites: the pancreas, Brockman body, and salivary gland (Fig 4D–4F). In the case of OlTGK2, apparent staining was obtained in the regions of the pharyngeal teeth (Fig 5D) and intervertebral disk (Fig 5E).

**Fig 6:**
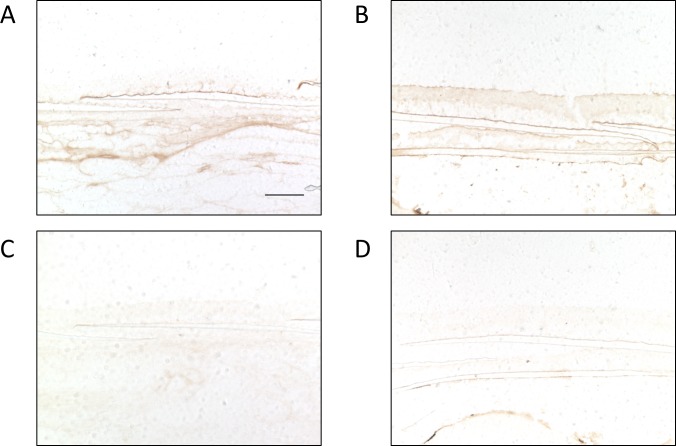
Immunostaining for OlTGK1 and OlTGK2 in the skin epidermis. Serial frozen tissue sections prepared and without fixation were subjected to immunohistochemical analysis using polyclonal antibodies as described in the legend to Figs 4 and 5. The analysis focused on areas of the epidermis: OlTGK1 (A), OlTGK2 (B), and negative controls (C, D) are shown. Scale bar indicates 100 μm.

Unexpectedly, in each preparation, less signal was observed in the skin epidermis of medaka than those in mammals in the previous results. We speculated the skin may have been degraded during the fixation procedure, which used acid-alcohol. Therefore, sections were prepared without any fixation treatment. These sections were subjected to immunoreaction using the same procedure employed with the fixed sections. As a result, apparent signals were obtained in the skin epidermis in immunodetections for both OlTGK1 and OlTGK2 (Fig 6). The epidermis is located above the squama in fish; the stained areas are shown along the layered epidermis. However, different areas were stained for each isozyme, indicating that expression of two enzymes was distinct.

## Discussion

Medaka have been used as a model fish, in parallel to zebrafish, for a wide range of applications in biology. Since investigation of TGs has not been carried out in this model fish, we started by characterizing the TG orthologues in this study. Through search of the medaka cDNA database and also genome database, seven corresponding genes were eventually identified and six were assigned as an orthologue to each human isozyme under the reasonable identity (%) of the amino acid sequence: FXIII (OlTGB; 47%), TG1 (OlTGK1; 55%, OlTGK2; 50%, OlTGK3 50%), TG2 (OlTGT; 55%), and TG4 (OlTGF; 30%). However, in order to conclude these genes are the orthologues to human isozymes, it is essential to analyze whether each medaka TGs has functional identity to those of human enzyme.

As shown in the alignment, the expected TG characteristics were preserved in this organism, such as the catalytic triad (Fig 1B, His, Asp, Cys at the active site), possible calcium binding site, and a possible tertiary structure as containing four separate domains. Among the orthologues, for TG1, the skin-type TG in mammals, medaka had three distinct genes that are located close to each other in the phylogenetic tree. It was unexpected that TG1 was the only TG to have three orthologues although apparent orthologues corresponding to the other skin TGs such as TG3 and TG5 have not been found in medaka.

The zebrafish, as another model organism, has also recently been investigated to determine the organization of its corresponding TG genes [16]. In this fish, twelve genes encoding TG orthologues were found, although their respective physiological roles are unknown. Considering the genome size of the zebrafish (1700 Mb), smaller numbers of medaka TGs might be expected, given that the smaller size of medaka genome (700 Mb) [12, 13]. TG may play similar physiological roles, in both organisms, but there is little information available regarding this issue. In the study of zebrafish TGs, induction of unusual enzymatic activities resulted in aberrant bone formation and Wnt signaling, but it remains unclear which isozyme is responsible for the phenotype [16, 17].

In our study of medaka, we focused on orthologues of TG1, two of which showed elevated expression. Bacterial expressions of recombinant gene products as an active enzyme were successful by cultivation at lower temperature in both cases; this result could not be attained in the case of recombinant mammalian TG proteins (data not shown). By preparing sufficient quantities of the purified proteins, we characterized their enzymatic properties. As expected, both enzymes demonstrated calcium-dependent catalytic activity. There is similarity in the enzymatic activity of the two purified proteins, suggesting that these enzymes contribute equally to physiological events.

In previous studies, TG1 was found not only in the epidermal keratinocytes but also in the epithelium of various tissues such as the kidney, liver, and intestine [7, 9]. Beneath the plasma membrane of the terminally differentiated keratinocytes, cross-linked products consisting of several structural proteins, called the cornified envelope, are formed by TG catalyzed reactions. Four TGs such as TG1, TG3, TG5, and TG6 are cooperatively involved in construction of this supermolecule responsible for barrier function. In medaka, as expected, expression of OlTGK1 and OlTGK2 were observed above the squama, suggesting that both enzymes contribute to the formation of the epidermis. Because these enzymes are expressed in distinct areas, they might be implicated in distinct roles for cross-linking different substrates. However, since a structure of similar mammalian cornified envelope has not been confirmed, apparent contribution on epidermis formation of these orthologues remains unknown.

Surprisingly, highly specific expression of the enzyme, OlTGK1 and OlTGK2 was observed in tissues other than the skin epidermis where we had expected it to be mainly expressed. OlTGK1 expression was observed in the pancreas, salivary gland, and Brockman body, which are common secreting tissues, although the physiological relevance remained unknown [22]. OlTGK2 expression was observed in the pharyngeal teeth and the intervertebral disk in addition to the surface of the body. The pharyngeal teeth and intervertebral disk may play roles in the digestion of food prior processing in stomach and in the integrity of the spine (backbone), respectively. Expression of OlTGK2 in the pharyngeal teeth was notable, since mouse TG1 was expressed in the teeth and forestomach as epidermal-like structure [23]. The precise significance of these observations is still unclear, but identification of the OlTGKs substrates in these tissues might provide some hints. In mammals, TG1 is found in other tissues than the epidermis. Indeed, TG1 plays a protective role during oxidative stress in kidney [24]. Although much less information is available on this issue, loss- or gain of function analysis will provide clue that may help to clarify the physiological significance of such expression. Double/ triple gene-depletion analyses of skin TG-orthologues in medaka might contribute to such investigations. We are currently working on this project and hope to be able to report results elsewhere in the future.

## Supporting Information

S1 Fig RT-PCR analysis of OlTGK1, OlTGK2, and OlTGK3. Medaka cDNA was synthesized using whole-body total RNA and used as a template to amplify the orthologues for human TG1. Primers for each reaction were designed to amplify the fragments: OlTGK1, OlTGK2, and OlTGK3. EF-Tu was used as a positive control. The DNA ladder marker was paralleled. Primers sequences: OlTGK1; CTGAAGGTGTGCTCAGTGGAC (Forward) and TCTTGTCCGGATTGTGAGTG (Reverse), OlTGK2; ACAGAACATCACACGGACCTC (Forward) and CAGGGGTTGAAGAGCATGTAG (Reverse), OlTGK3; GTAAGCCTGAACAGCCAGCTGG (Forward) and CAACACCGTGAACCTG (Reverse), EF-Tu; CAGGACGTCTACAAAATCGG (Forward) and AGCTCGTTGAACTTGCAGGCG (Reverse).
(TIF)

S2 Fig Expression of recombinant proteins (OlTGK2) in bacteria cultured at different temperatures. The soluble (sup) and insoluble (ppt) fractions of an extract of the bacteria that express recombinant OlTGK2 protein were loaded. Samples were prepared from cells in which overexpression was induced at 37°C (left) and 26°C (right). The molecular mass marker was paralleled.
(TIF)

S3 Fig Polyclonal antibodies against OlTGK1 and OlTGK2 do not cross-react. Recombinant OlTGB, OlTGT, OlTGO, and OlTGF proteins were prepared using the procedure described for the OlTGKs (unpublished data). Immunoblot analysis of these proteins was performed using polyclonal antibody against OlTGK1 and OlTGK2. Similar amounts (10 ng) of each purified recombinant protein was loaded and then blotted for immunoreaction. The blots were probed with affinity-purified polyclonal antibodies against OlTGK1 (A) and OlTGK2 (B) and then developed using the chemiluminesence.
(TIF)

## Abbreviations

bio-Cd: biotin-labeled cadaverine
PBS: phosphate-buffered saline
TBS: Tris-based buffered saline
