# Supplementary figures and images for: Biochemical Characterization of Medaka (Oryzias latipes) Transglutaminases, OlTGK1 and OlTGK2, as Orthologues of Human Keratinocyte-Type Transglutaminase
Source: PLoS One. 2015 Dec 29;10(12):e0144194. doi: 10.1371/journal.pone.0144194 (PMC4694659; doi:10.1371/journal.pone.0144194)

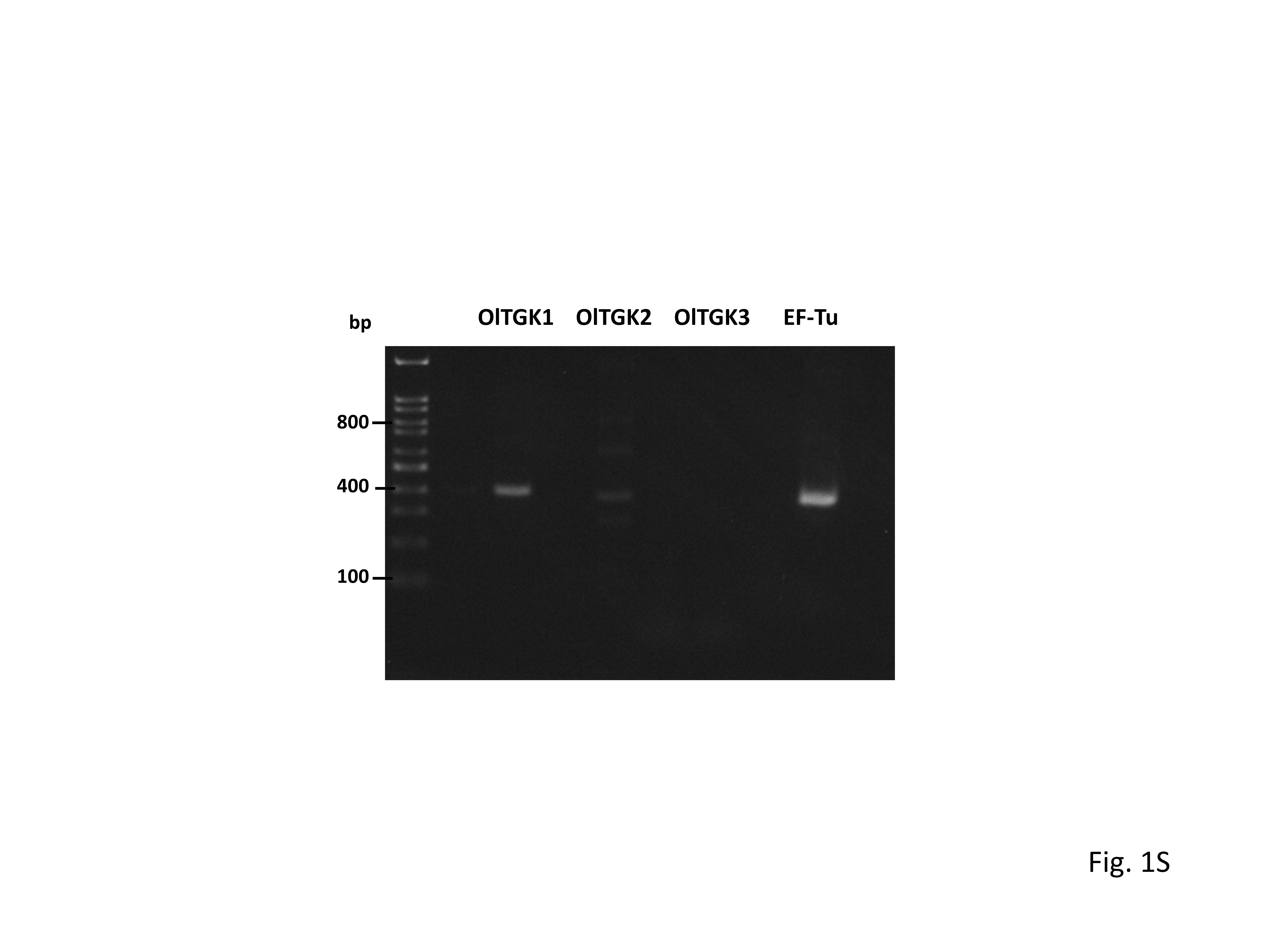

Supplement: S1 Fig — Medaka cDNA was synthesized using whole-body total RNA and used as a template to amplify the orthologues for human TG1. Primers for each reaction were designed to amplify the fragments: OlTGK1, OlTGK2, and OlTGK3. EF-Tu was used as a positive control. The DNA ladder marker was paralleled. Primers sequences: OlTGK1; CTGAAGGTGTGCTCAGTGGAC (Forward) and TCTTGTCCGGATTGTGAGTG (Reverse), OlTGK2; ACAGAACATCACACGGACCTC (Forward) and CAGGGGTTGAAGAGCATGTAG (Reverse), OlTGK3; GTAAGCCTGAACAGCCAGCTGG (Forward) and CAACACCGTGAACCTG (Reverse), EF-Tu; CAGGACGTCTACAAAATCGG (Forward) and AGCTCGTTGAACTTGCAGGCG (Reverse). (TIF) [file pone.0144194.s001.tif]

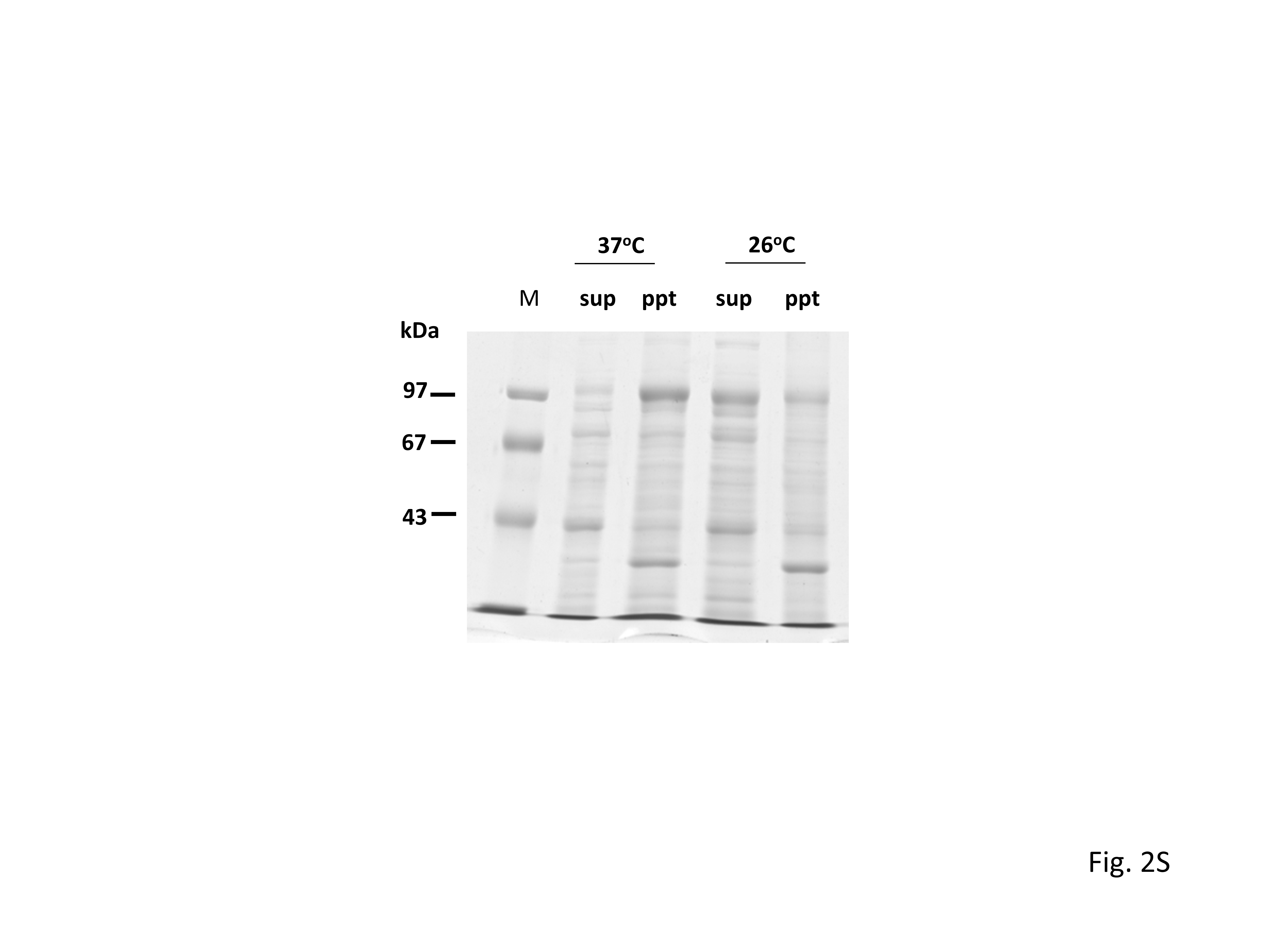

Supplement: S2 Fig — The soluble (sup) and insoluble (ppt) fractions of an extract of the bacteria that express recombinant OlTGK2 protein were loaded. Samples were prepared from cells in which overexpression was induced at 37°C (left) and 26°C (right). The molecular mass marker was paralleled. (TIF) [file pone.0144194.s002.tif]

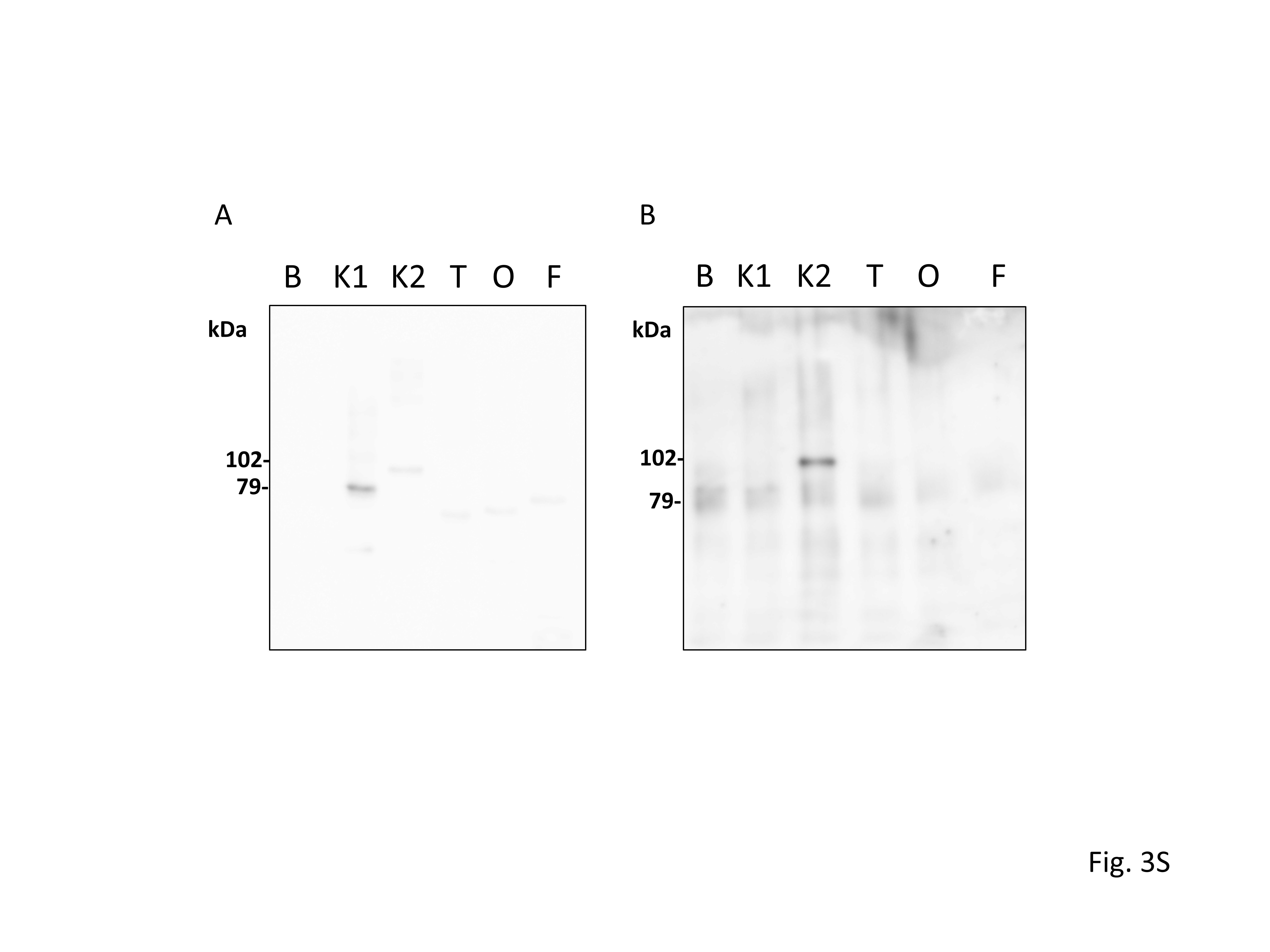

Supplement: S3 Fig — Recombinant OlTGB, OlTGT, OlTGO, and OlTGF proteins were prepared using the procedure described for the OlTGKs (unpublished data). Immunoblot analysis of these proteins was performed using polyclonal antibody against OlTGK1 and OlTGK2. Similar amounts (10 ng) of each purified recombinant protein was loaded and then blotted for immunoreaction. The blots were probed with affinity-purified polyclonal antibodies against OlTGK1 (A) and OlTGK2 (B) and then developed using the chemiluminesence. (TIF) [file pone.0144194.s003.tif]
